# Mirror and Vibration Therapies Effects on the Upper Limbs of Hemiparetic Patients after Stroke: A Pilot Study

**DOI:** 10.1155/2018/6183654

**Published:** 2018-11-04

**Authors:** Maria da Conceição Barros Oliveira, Danylo Rafhael Costa Silva, Bruno Vieira Cortez, Constância Karyne da Silva Coêlho, Francisco Mayron de Sousa e Silva, Giselle Borges Vieira Pires de Oliveira, Danúbia de Cunha de Sá-Caputo, Angela Cristina Tavares-Oliveira, Mário Bernardo-Filho, Janaína De Moraes Silva

**Affiliations:** ^1^Federal University of Piauí, Post-Graduation of the Northeast Biotechnology of the Federal University of Piauí, Teresina, PI, Brazil; ^2^Mauricio de Nassau Faculty, Department of Physiotherapy, Teresina, PI, Brazil; ^3^State University of Piauí, Department of Physical Therapy, Teresina, PI, Brazil; ^4^University of the State of Rio de Janeiro, Post-Graduate Program in Medical Sciences, Rio de Janeiro, RJ, Brazil; ^5^University of the State of Rio de Janeiro, Roberto Alcantara Gomes Institute of Biology, Department of Biophysics and Biometrics, Laboratory of Mechanical Vibrations and Integrative Practices, Rio de Janeiro, RJ, Brazil

## Abstract

**Background/Aim:**

To evaluate, in this pilot study, the effects of the mirror (MT) and vibration therapies (VT) on the functionality of hemiparesis patients after stroke.

**Materials and Methods:**

Twenty-one individuals after stroke with upper limb hemiparesis were randomized into control group (CG), Mirror Therapy Group (MTG), and Vibration Therapy Group (VTG). The functionality was evaluated before and after 12 sessions with three tests (i) Mobility Index Rivermead, (ii) Motor Function Wolf Test (time, functional ability), and (iii) Jebsen Taylor Test.

**Results:**

Significant findings were observed for MTG or VTG when compared to the CG, obtaining improvements in the three functional tests: Mobility Index Rivermead, Motor Function Test Wolf (time) and Motor Function Test Wolf (functional ability), and Jebsen Test Taylor.

**Conclusions:**

MT or VT showed enhancements on the functionality of subjects with poststroke hemiparesis. In consequence, these interventions may be used in the rehabilitation of these individuals in order to promote improvements of the affected upper limb functionality. Probably, neuromuscular responses of the used therapies would be related to these desirable effects. However, it is necessary conducting further controlled studies with more subjects.

## 1. Introduction

Stroke remains one of the most undesirable and devastating of the neurological diseases. It can be defined as a clinical syndrome that develops rapidly signs of focal or global disturbances of the cerebral function with vascular origin, with symptoms that persist for longer than 24 hours. It is often responsible for the death and for several gross physical limitations, restrictions, or disabilities [1, 2]. The alteration of the functionality of the upper limbs (UL) is one of the highest complaints of individuals with stroke due to the limitations in performing important manual daily activities [3]. Therefore, the use of rehabilitation therapies to try to restore the functionality of these patients [4] is relevant, and among these are the mirror therapy (MT) [5] and the vibration therapy (VT) [6].

MT explores the effects obtained by the visual perception through a mirror of a determined movement [4, 7, 8]. This provides to the individual an appropriate visual stimulus, which hypothetically may consist of a strategy of “motor copy” to the affected limb. The external feedback with use of a mirror and an internal feedback with mental practice of functional activities are used [9, 10]. The stimulus generated by an intact sensory region can be utilized to access and recruit specific neural circuits that are dormant in other brain regions. In consequence a neural network, which would be responsible for the control of a hand in a certain task, can be used to control the other hand [11]. Therefore, studies suggest that MT can aid to accelerate the functional recovery of a wide range of sensorimotor disorders, including poststroke hemiparesis [12–15].

VT uses a device that transmits mechanical vibration throughout the whole or part of the body [16–18]. VT promotes improvements in muscle strength [17], cardiovascular parameters [19] and bone mineral density [20], functional capacity [21], sensorimotor integration [22], and electrophysiological changes [23]. In addition, VT acts in the musculoskeletal system promoting the synchronization of the motor units and improves the synergism between the agonist and antagonist muscles [24, 25]. VT also seems to generate the tonic vibration reflex that occurs due to the increase of the afferent inflow of the primary muscle spindle [22, 26]. This fact indicates that the projections for the somatosensory cortex can modulate the excitability of the motor cortex, reinforcing the hypothesis that vibratory stimulus influences the cortical responses [27, 28].

Putting together the findings reported [5, 29–31] subjects with poststroke hemiparesis may have potential benefits due to the use of MT and VT. In consequence, the (i) MT would promote activation in the cortical visual area and in the areas involved in motor behavior [32] and (ii) the VT would promote normal motor activity patterns by modulating the excitability of motor neurons and corticospinal tract [33].

Considering the clinical relevance of the MT and of the VT and the limitations of the individuals with stroke, this pilot study aimed to evaluate effects of MT and of VT on the functionality individuals with poststroke hemiparesis.

## 2. Material and Methods

*Ethics Approach and Selection of the Participants*. This study was carried out from August up to December 2014. The selected participants were performing physical therapy in the Clinical School of the* Faculdade Maurício de Nassau*, Teresina, PI, Brazil.

The study was approved by the Ethics Committee of the* Hospital São Marco *with the number 722718. The principles embodied in the Declaration of Helsinki were followed.

### 2.1. Inclusion Criteria

The study included individuals with hemiparesis of upper limb (UL) aged between 45 and 75, both genders, diagnosis of stroke with the minimum of 12 months of sequel or spastic phase established, absence of cognitive limitations, spasticity 1, 1+ and 2 on the modified Ashworth Scale [15, 34], and lack of orthopedic disorders in UL paretic.

### 2.2. Exclusion Criteria

The exclusion criteria considered the individuals with cardiorespiratory, dysphasia, or Wernicke's aphasia, in use of muscle relaxants and with contraindications for use of vibration referred to the used equipment. The individuals who refused to sign the consent form for participation in the study were also excluded.

### 2.3. Convenience Sampling

The individuals that have participated in the study, as a convenience sampling, were enrolled according to their availability and accessibility.

### 2.4. Intervention

The interventions and data collection were carried out by a single evaluator following the availability of individuals. A sequence of three sessions per week, totaling twelve visits, was carried out.

Twenty-one individuals after stroke hemiparesis in the UL were randomized into three groups, control group (CG, n = 7), Mirror Therapy Group (MTG, n = 7), and Vibration Therapy Group (VTG, n = 7).

The subjects of CG held conventional physiotherapy for the rehabilitation of stroke.

The individuals of VTG underwent 15 minutes of uninterrupted vibratory therapy with the contact with a Digital Vibration Pad (Nissan Fisio, São Paulo, Brazil). The frequency used was 35 Hz, the amplitude 1.5mm, and the intensity 3. The subjects were seated in a chair with UL paretic naked and relaxed on the vibrating pad.

The individuals of the MTG underwent a bimanual activities of a protocol (2 sets of 10 repetitions with (i) flexion and extension of the shoulder, elbow and wrist, (ii) abduction and adduction of the shoulder, (iii) pronation and supination of the elbow, (iv) flexion with horizontal abduction and flexion with horizontal adduction of shoulders, and (v) flexion and extension of the elbow in supine position with the palm of closed hand and with opponency of the fingers, drawing in the air a circle, a triangle, a square, and a rectangle, in the seated position on a chair. On this chair was placed a laying mirror interposed laterally between its upper and lower front of the chest. Subjects were instructed to observe the health limb through the reflection of the mirror and to perform the same activities with the paretic limb.

### 2.5. Evaluations

All the individuals were evaluated before and after the intervention of each group, considering (i) the upper limb motor function with the motor function of the Wolf Test [35], (ii) the mobility of the upper border with the Rivermead Mobility Index [36], and (iii) manual function with the manual function using the Jebsen Taylor Test [37]. Lennon and Johnson [36] have considered that the Rivermead Mobility Index is essential for demonstrating treatment effects in individuals following a stroke.

### 2.6. Statistical Analysis

Statistical analysis was performed using the SPSS software, version 18.0. Quantitative variables were presented with descriptive statistics (mean, standard deviation). Qualitative variables were presented as ratio.

Shapiro-Wilk test was firstly used to assess the normality of the quantitative variables. To analyze differences between the values of the Rivermead Mobility Index, manual function with the Jebsen Taylor Test Manual and the motor function with the Wolf Test used the Wilcoxon test for two averages and the Kruskal-Wallis with post-hoc Dun to three or more averages. In all analyzes, the statistical level of significance of 95% (*p*<0.05) was considered.

## 3. Results

Among the 21 individuals, 13 were females and 8 were males. The mean age was 60.1 years, with the minimum and maximum values of 55 and 65, respectively.

**Figure 1:**
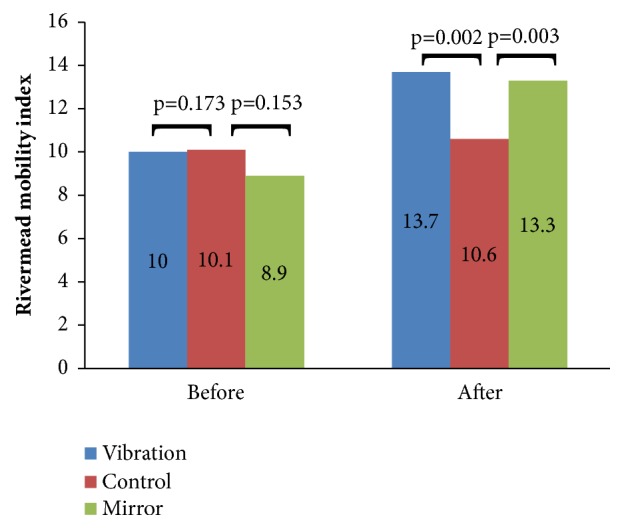
Data of the Rivermead Mobility Index among the individuals of the control group, vibration and mirror therapy before and after the interventions.

In Figure 1, it is possible to verify that when comparing the VTG or MTG to CG before the intervention, there were no changes regarding the parameters of the Rivermead Mobility Index. However, after the intervention there was a significant improvement in the individuals of MTG (*p* = 0.003) and of VTG (*p* = 0.002) compared to the CG.

**Figure 2:**
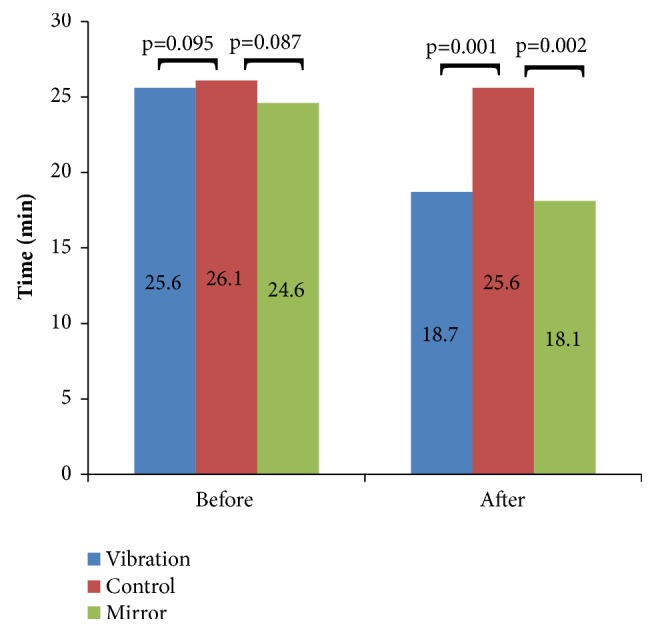
Data of the* Jebsen Taylor Test* among the individuals of the control group, vibration and mirror therapy before and after the interventions.

Figure 2 shows the comparative data of the Jebsen Taylor Manual function test between the control group, vibration or mirror therapy before and after the interventions. No changes among the groups before the intervention were verified. However, after that there was a significant improvement in the individuals of the MTG (*p *= 0.002) or of the VTG (*p* = 0.001).

**Figure 3:**
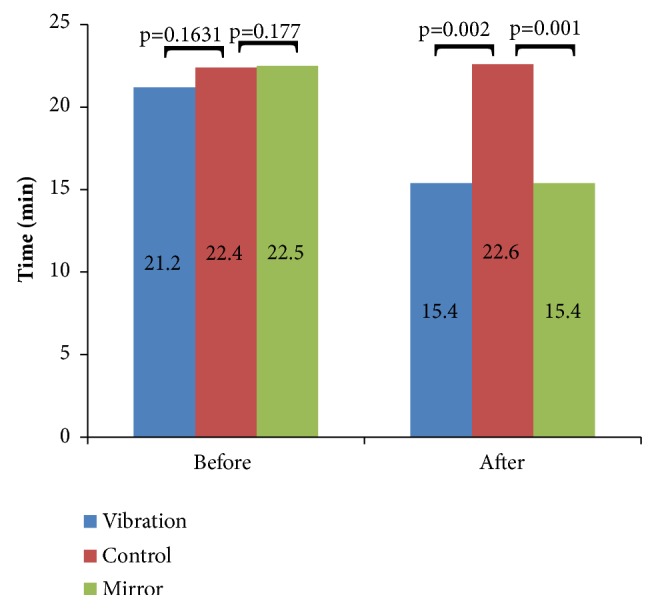
Data of the motor function (time) among the individuals of the control group, vibration and mirror therapy before and after the interventions.

Figure 3 shows the comparative data of motor function of the Wolf Test (Time) of the control group, vibration and mirror therapy before and after the interventions. It is verified that after the intervention there was a significant improvement in the MTG (*p* = 0.002) or in the VTG (*p* = 0.001) when compared to the CG.

**Figure 4:**
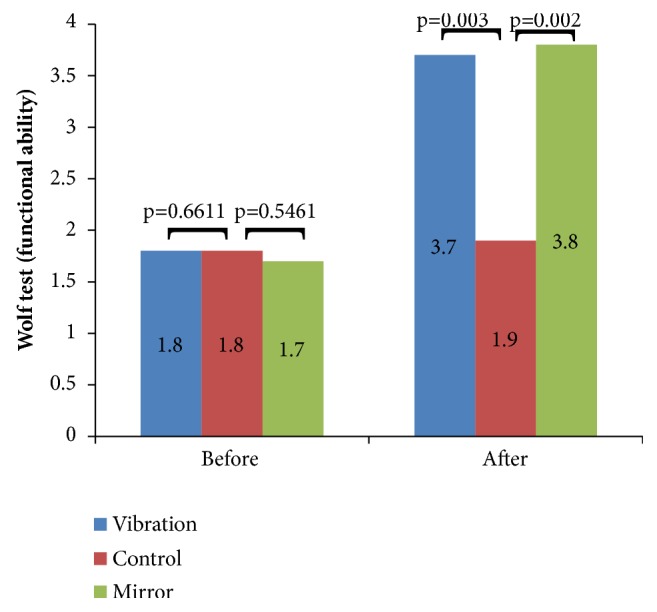
Comparative data of motor function of the Wolf Test (functional ability) of the control group, vibration and mirror therapy before and after the interventions.

Figure 4 shows the comparative data of motor function of Wolf Test (functional ability) of the CG, and the participants of the groups that have performed vibration therapy and mirror therapy. It is observed a significant improvement in individuals of the MT (*p* = 0.002) or VTG (*p* = 0.003) in comparison with the CG.

## 4. Discussion

Piassaroli et al. [38] have reported that the incidence of stroke is higher in females, and the mean age of onset of stroke was 60 years. Considering this statement, the individuals of the current work had an average of 60.1 years and mostly were females.

The significant results, of this pilot study, on the functionality of individuals following use of MT (Figures 1, 2, 3) are in agreement with Altschuler et al. [39]. Probably, the MT influenced the motor recovery of amplitude, speed, and accuracy of the movements of affected UL, aiding the improvement of its functionality. These positive effects were due to the MT contribute to provide an appropriate visual stimulus of the patient, possibly replacing the proprioception, which often is reduced or absent [40].

The efficacy of the MT in poststroke patients with little or no upper limb motor function has been evaluated in several works with positive results [4, 39, 41]. Positive results were also found in the current study (Figures 1, 2, 3). In randomized controlled investigations [42, 43] significant improvement in function in patients with poststroke hemiparesis is presented. Both studies used 40 patients with hemiparesis, one with patients with hemiparesis of the lower limbs [41] and the other with patient with hemiparesis of lower limbs enrolled up to 12 months after stroke [41]. In these studies [41, 44], subjects were randomized into MT or control group, and all subjects received a physical therapy protocol as a control intervention. It was then observed a significant improvement of the sensorimotor deficits by the Brunnstrom scale and by the Physical Performance Scale Fugl-Meyer in participants that have performed the MT in comparison with the participants of the control group.

The VT also produced significant improvements on the functionality of stroke individuals (Figures 1, 2, 3). These data are in agreement with Shinohara et al. [45] that showed by electromyographic data, after 30 minutes of VT on the tendons of the flexors of handle of healthy individuals, an increase of activity corticospinal muscle short radial extensor. The use of the VT on spastic patients provided a reduction of spasms and a muscle relaxation influencing positively the function of the affected limb. Other studies [6, 46] have also demonstrated significant effects in the functionality after the use of the VT. Costantino et al. [6] in a study with 32 poststroke patients with spasticity of the upper limbs verified that VT presented statistical improvements in these patients in the muscle strength and quality of life and reduction of the pain and spasticity. Cordo et al. [46] evaluated 20 spastic patients after VT and observed an improvement of the motor condition of the subjects related to the range of movement and stability of the walking, which lasted for about 6 months after the intervention.

Despite the promising results, the current study has some limitations. It should be noted that the population of the current study consisted of outpatients. Moreover, a relatively small number of subjects have participated of this pilot work.

Considering the findings, the use of the MT or of the VT has shown positive effects on the functionality of hemiparesis after stroke, it is concluded that these interventions would be useful in the rehabilitation of hemiparetic stroke individuals. Probably, neuromuscular responses of the used therapies would be related to the improvements of the affected upper limb functionality. Despite the results, however, there is the recommendation that is necessary to carry out controlled studies with larger samples to strengthen the evidence base of the application of simple and useful techniques to the management of individuals with stroke.

## Data Availability

The data used to support the findings of this study are available from the corresponding author upon request.
